# TRPV4 Stimulation Induced Melatonin Secretion by Increasing Arylalkymine *N*-acetyltransferase (AANAT) Protein Level

**DOI:** 10.3390/ijms18040746

**Published:** 2017-04-01

**Authors:** Hanan Awad Alkozi, Maria J. Perez de Lara, Juan Sánchez-Naves, Jesús Pintor

**Affiliations:** 1 Department of Biochemistry and Molecular Biology IV, Faculty of Optics and Optometry, University Complutense of Madrid, 28040 Madrid, Spain; Hanan-q1@live.com (H.A.A.); mariajpdl@opt.ucm.es (M.J.P.d.L.); 2 Department of Ophthalmology, Balear Institut of Ophthalmology, 07011 Palma de Mallorca, Spain; juansanchez.naves@gmail.com

**Keywords:** AANAT, ciliary body, eye, melatonin, TRPV4

## Abstract

Melatonin is a molecule which has gained a great deal of interest in many areas of science; its synthesis was classically known to be in the pineal gland. However, many organs synthesize melatonin, such as several ocular structures. Melatonin is known to participate in many functions apart from its main action regulating the circadian rhythm. It is synthesized from serotonin in two steps, with a rate-limiting step carried out by arylalkymine *N*-acetyltransferase (AANAT). In this report, the role of TRPV4 channel present in human ciliary body epithelial cells in AANAT production was studied. Several experiments were undertaken to verify the adequate time to reach the maximal effect by using the TRPV4 agonist GSK1016790A, together with a dose–response study. An increase of 2.4 folds in AANAT was seen after 18 h of incubation with 10 nM of GSK1016790A (*p* < 0.001, *n* = 6). This increment was verified by antagonist assays. In summary, AANAT levels and therefore melatonin synthesis change after TRPV4 channel stimulation. Using this cell model together with human ciliary body tissue it is possible to suggest that AANAT plays an important role in pathologies related to intraocular pressure.

## 1. Introduction

Melatonin is an indolamine synthesized by several ocular structures apart from its classical production in the pineal gland. It is originally known to regulate the circadian rhythm, however, many studies have indicated further important functions of melatonin, such as its role as an antioxidant, antidepressant, suppressing carcinogenesis, among other functions [1,2,3,4]. Melatonin presence in the eye is fundamental since it participates in numerous functions such as controlling tear secretion [5], accelerating corneal wound healing [6], controlling intraocular pressure (IOP) and regulating retinal physiology [7,8]. All these actions are mediated by melatonin membrane receptors whose presences have previously been described in the eye [9].

Melatonin is well known for following a circadian rhythm, which has higher levels during the night and lower levels at daytime [10]. This pattern matches with the changes observed in IOP, as when melatonin levels rise at night, intraocular pressure comes down [11]. This observation opened a window of investigation to understand the link between IOP and melatonin.

One of the leading causes of irreversible vision loss is glaucoma, a multifactorial optic neuropathy that results in progressive blindness. The only risk factor that can be controlled in glaucoma is the elevated intraocular pressure. Studies have shown that melatonin and its analogs are able to bring down IOP by exogenous consumption [12,13]. Surprisingly, a recent study analyzing melatonin levels in human aqueous humors has demonstrated that those patients with elevated intraocular pressure present higher melatonin concentrations than healthy subjects [14,15]. These patients should have lower IOP, nonetheless this does not occur. The reasons that cause it are not yet understood.

Melatonin is synthesized from serotonin through two steps. In the first, serotonin is transformed to *N*-acetylserotonin (NAS) through acetylation by an enzyme called arylalkymine *N*-acetyltransferase (AANAT). This enzyme catalyzes the transfer of acetyl group from acetyl-CoA to serotonin. In the second step, to convert NAS to melatonin, the second enzyme called hydroxyindole-*O*-methyltransferase (HIOMT) is responsible for the *O*-methylation [16,17].

The first enzyme in the melatonin synthesis, AANAT, seems to be the key enzyme regulating melatonin synthesis. Studies have shown that AANAT fluctuate following a circadian rhythm [18,19,20], while HIOMT does not seem to change [21]. In fact, this is critical given that melatonin changes throughout the day; however, it is possible that AANAT is regulated by other environmental factors such as hormones, food or drug intake [22,23,24]. AANAT seems to have two ways of regulation. One is a quick process to protect the enzyme against degradation that happens through its phosphorylation. This regulatory mechanism also depends on a protein termed 14-3-3 that binds to AANAT and which has been linked to the activation of PKA after cAMP generation [25]. The second regulation mechanism is a long-term one, which is also dependent on cAMP/protein kinase A pathway but which activates gene expression. In rodents, transcriptional activation of *aanat* gene is the classical mechanism to induce melatonin biosynthesis. It involves PKA-dependent phosphorylation of the transcription factor cyclic AMP response element binding protein (CREB) [26] and binding of phosphorylated CREB in the promoter region of *aanat* gene.

Very recently, a transient receptor potential vanilloid 4 (TRPV4), a non-selective cation channel that regulates osmo-, thermo-, mechanosensation was said to play an important role in the ciliary body epithelium cells [27,28]. This channel activation has led to an increment of the extracellular level of melatonin [29]. These findings are pharmacologically relevant in the search of new therapies for glaucoma because melatonin has the ability to lower IOP as previously commented. In this report, we describe the effect of TRPV4 stimulation on the protein levels of AANAT, one of the enzymes responsible for melatonin synthesis, as well as its changes in the ciliary body of normal and glaucomatous patients.

## 2. Results

### 2.1. Presence of AANAT in the Human Ciliary Body

**Figure 1:**
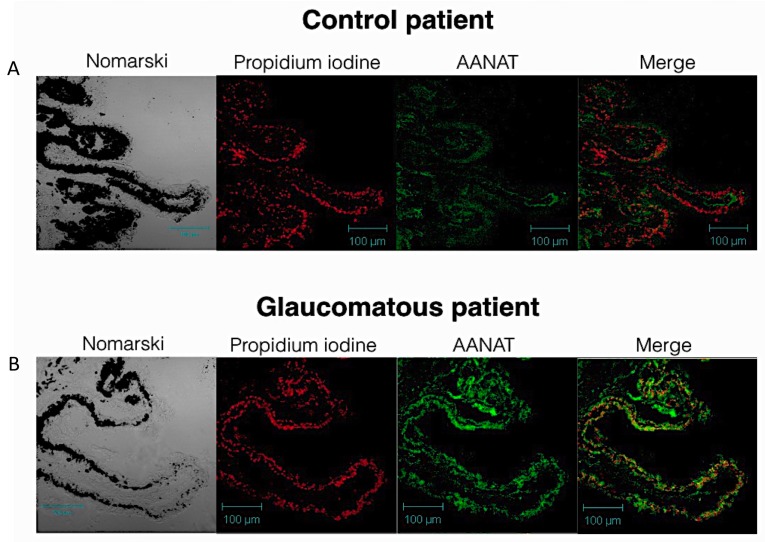
Apparent changes of AANAT in human ciliary body tissue: (**A**) Representative pictures of human ciliary processes (*n* = 2) of a non-glaucomatous individual (*n* = 4). From left to right, Differential Interference Contrast (DIC) image, nuclei (in red, propidium iodine), AANAT (in green) and merge image; (**B**) Representative image of human ciliary processes of a glaucomatous individual. From left to right, DIC image, nuclei (in red, propidium iodine), AANAT (in green) and merge image.

Human eyes were first treated for immunflourescent labeling, and the search for possible changes in the AANAT labeling in the ciliary body was undertaken by analyzing samples of ciliary body tissue of healthy subjects and comparing them to glaucomatous donors. Ciliary body epithelium presented a positive labeling in both normal and glaucomatous human samples (Figure 1).

**Figure 2:**
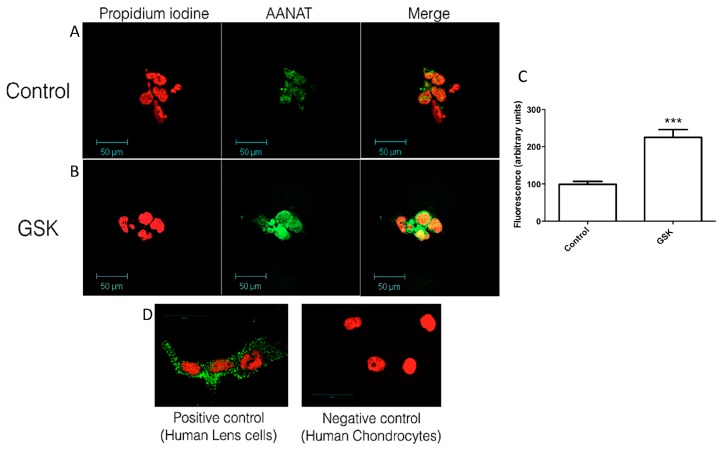
Presence and changes of AANAT in human ciliary body epithelial cells: (**A**) Untreated human ciliary body epithelial cells showing the expression of AANAT (in green) and the nuclei (in red); (**B**) Human ciliary epithelial cells after treatment with 10 nM GSK1016790A for 18 h. AANAT expression can be seen in green while nuclei appear in red; (**C**) Fluorescence quantification of the images shown in **A** and **B** for the AANAT intensity (green), normalized to control values; (**D**) Positive and negative controls for AANAT performed with human lens epithelial cells (positive) and human chondrocytes (negative). The values are the mean ± SEM of six independent experiments (*** *p* < 0.001).

In particular, a stronger fluorescent labeling was observed in the glaucomatous patient sections (Figure 1B *n* = 4) when compared to normal samples (Figure 1A, *n* = 2). This elevation in the expression of AANAT, in the case of the glaucomatous donors, was “in vitro” established using human ciliary body epithelial cells which were stimulated by the TRPV4 agonist GSK1016790A, as previously described [29]. The results obtained with the treated cells were consistent with the human ciliary body sections obtained from the donors. In this sense, the presence of AANAT was detected in both control and treated cells (Figure 2), the labeling being stronger in the GSK-treated cells (Figure 2B), than in the untreated cells (Figure 2A). Positive and negative controls were also performed for AANAT with human lens epithelial cells and human chondrocytes, respectively (Figure 2C) [30].

### 2.2. TRPV4 Activation Increases AANAT Protein Expression in Ciliary Body Epithelial Cells

**Figure 3:**
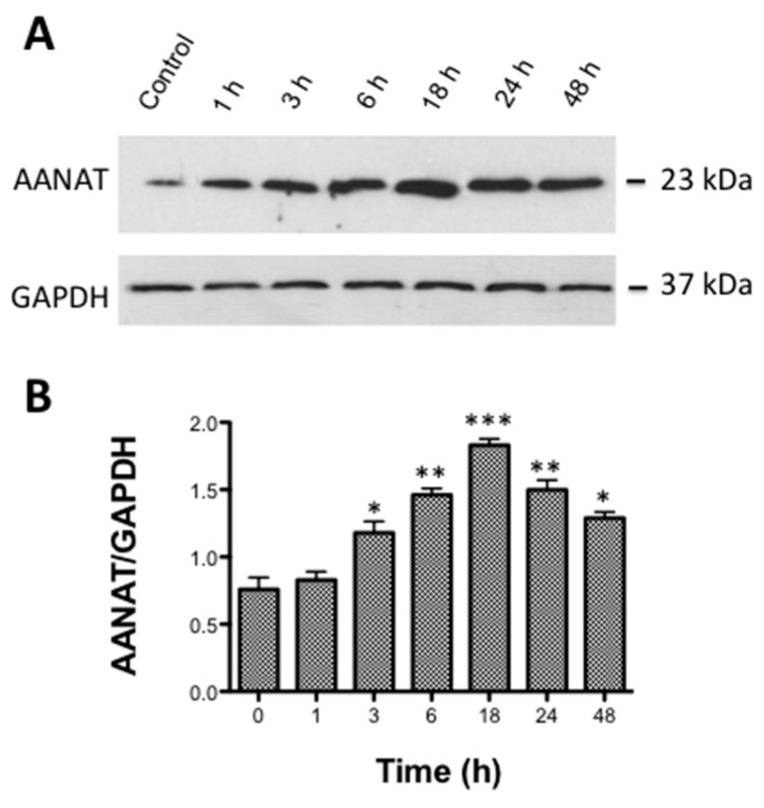
Time-course of the effect of GSK on AANAT protein synthesis: (**A**) representative Western blot showing the changes in AANAT during a maximal period of 48 h after cell treatment with 10 nM GSK1016790A; and (**B**) column plot showing the relative quantification of the Western blots band intensities. Values represent the mean ± SEM of six independent experiments (* *p* < 0.05, ** *p* < 0.01, *** *p* < 0.001 versus time 0).

The application of the selective TRPV4 agonist GSK1016790A during different times up to 48 h at a single dose of 10 nM, showed changes in AANAT expression as observed in Figure 3. The results indicated that AANAT expression has a clear maximal peak of 2.4 folds above the control value after 18 h of incubation with TRPV4 agonist (Figure 3A, *** *p* < 0.001, *n* = 6).

Interestingly, the values of AANAT showed a decrease after that maximal expression of the enzyme, returning towards the initial levels (Figure 3B).

### 2.3. TRPV4 Activation Increases Melatonin Levels in Ciliary Body Epithelial Cells

**Figure 4:**
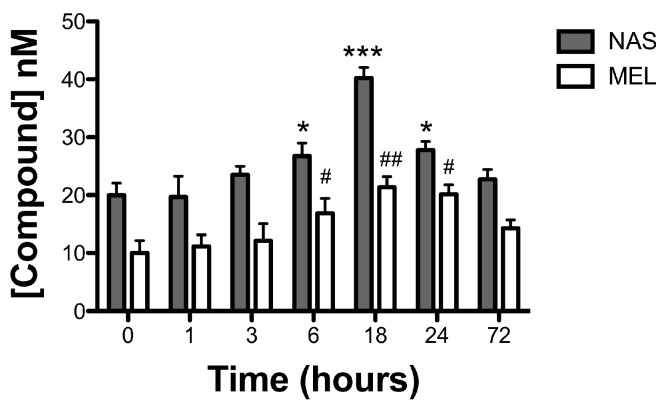
Time-course on the effect of GSK on NAS and melatonin levels. Columns presenting the concentrations of NAS and melatonin calculated as described in material and methods. Values represent the mean ± SEM of six independent experiments (* *p* < 0.05 and *** *p* < 0.001 versus time 0 for NAS and ^#^ *p* < 0.05 and ^##^ *p* < 0.01 versus time 0 for melatonin).

Since AANAT is responsible for melatonin synthesis by producing the precursor *N*-acetyl serotonin (NAS), HPLC studies were performed to investigate a possible correlation between the expression levels of AANAT showed in Figure 3 and the production of both, NAS and melatonin. As can be seen in Figure 4, both the levels of NAS and melatonin presented maximal concentrations at 18 h, with concentrations of 40.24 ± 1.82 nM in the case of NAS and 21.36 ± 1.83 nM for melatonin (*n* = 6, *** *p* < 0.001 vs. control for NAS and ^##^ *p* < 0.01 vs. control for melatonin). As happened in the case of AANAT expression, after 18 h, both NAS and melatonin returned to their initial values.

### 2.4. Concentration-Response Study of GSK Effect on AANAT Levels in Ciliary Body Epithelial Cells

**Figure 5:**
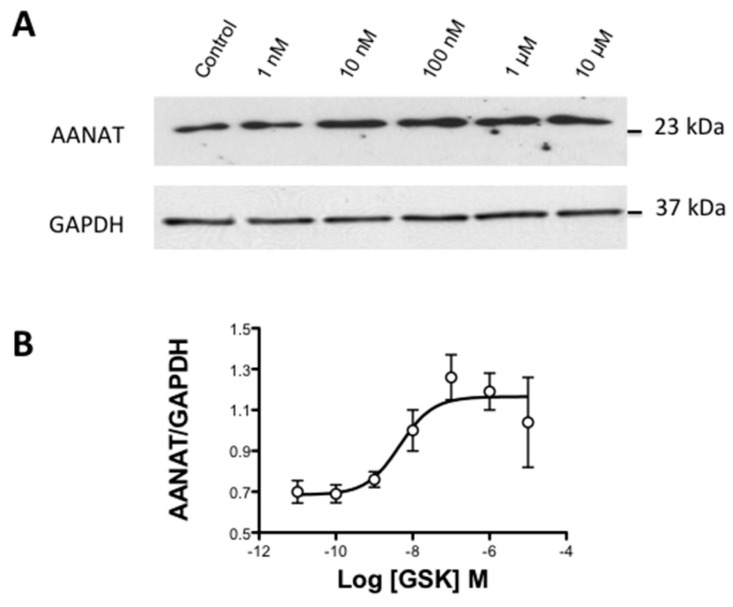
Concentration–response curve for GSK1016790A on AANAT phosphorylation level: (**A**) representative Western blot study showing the concentration dependency of AANAT phosphorylation when cells are challenged with GSK1016790A ranging from 1 nM to 10 M; and (**B**) concentration–response curve plotted with the relative quantification of the Western blot band intensities. The values represent the mean ± SEM of five independent experiments.

After adjusting the time necessary to reach the maximal AANAT expression when activating the TRPV4 channel, a concentration–response assay was performed at this time by applying different graded concentrations of GSK ranging from 1 nM to 10 µM. In this sense, it was possible to observe that AANAT expression reached a maximum at GSK concentration of 10^−8^ M (Figure 5A). Transformation of Western blots into a dose–response curve allowed the observation of a sigmoidal pattern that provided a pD_2_ value for GSK of 8.34 ± 0.30, which was equivalent to an EC_50_ value of 4.57 nM (*n* = 5, Figure 5B).

### 2.5. Effect of TRPV4 Antagonists on AANAT Levels in Ciliary Body Epithelial Cells

**Figure 6:**
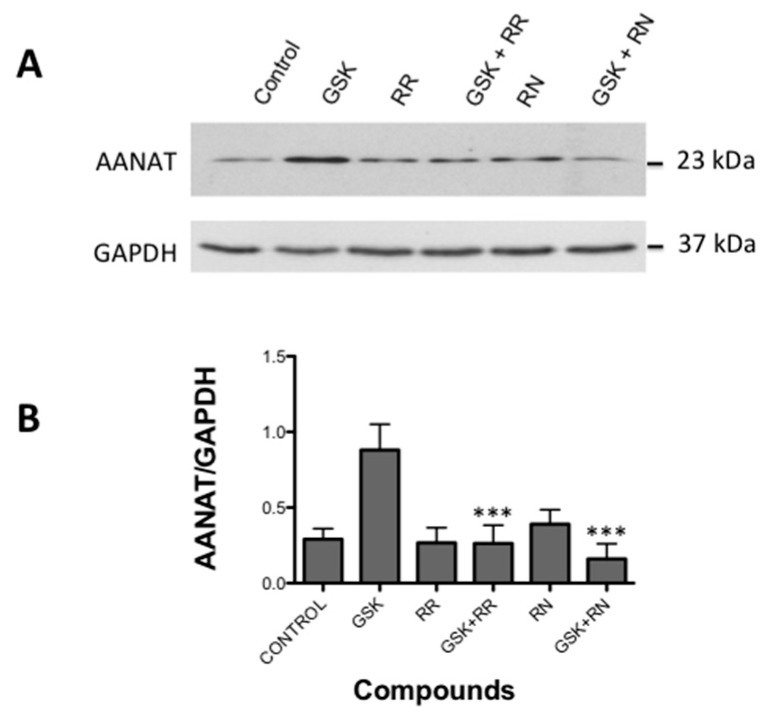
Effect of TRPV4 antagonists on AANAT phosphorylation triggered by GSK1016790A: (**A**) representative Western blot study showing the activity of TRPV4 antagonist Ruthenium Red (RR) and RN-1734 (RN), both alone and together with GSK1016790A, following the protocol described in methods; and (**B**) column plot showing the relative quantification of the Western blots band intensities. Values represent the mean ± SEM of five independent experiments (*** *p* < 0.001 versus GSK1016790A effect).

Different antagonists were used to confirm that the effect shown by GSK1016790A was actually acting on TRPV4 channel. The application of 10 nM of GSK after 18 h produced a significant increase in AANAT compared to non-treated cells (Figure 6A). This increment was blocked after applying a non-selective TRPV1/TRPV4 antagonist Ruthenium Red (RR) and by the selective antagonist of the TRPV4, RN-1734 (*p* < 0.001 for both compounds vs. GSK alone; *n* = 5, Figure 6B).

## 3. Discussion

**Figure 7:**
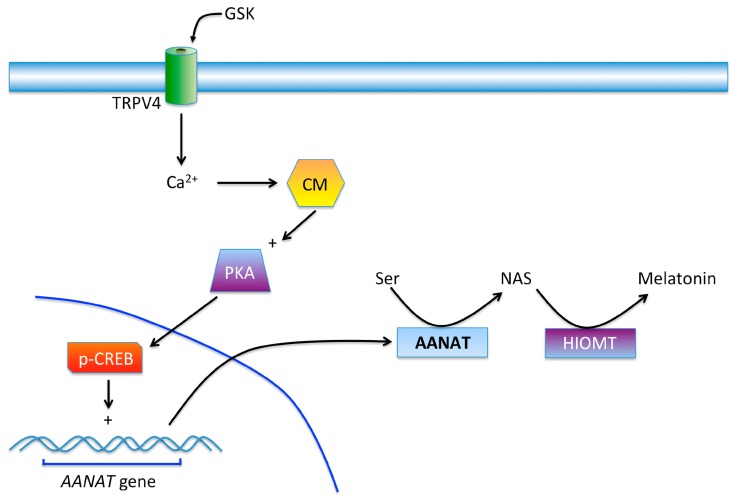
Possible mechanism of action of GSK acting on the TRPV4 channel in the ciliary body. After the activation of the TRPV4 the influx of Ca^2+^ will bind to calmodulin (CM) which activates adenylate cyclase and later protein kinase A (PKA). This kinase will finally produce the phosphorylation of CREB (cAMP response element-binding) than may stimulate the synthesis of the enzyme AANAT. This protein together with HIOMT will finally augment the production of melatonin in ciliary body epithelial cells. TRPV4 channel can be stimulated by the abnormal elevation in IOP that often occur in glaucoma pathology.

In this study, we have described that the TRPV4 channel stimulation in the ciliary body epithelial cells is able to increase the expression of the rate-limiting enzyme of melatonin synthesis aralkylamine *N*-acetyltransferase (AANAT) (Figure 7). This increment of AANAT is both time and dosage dependent, and reaches its maximal effect after 18 h of stimulation with the selective TRPV4 agonist GSK1016790A at 10 nM concentration [31]. This effect was blocked by TRPs antagonist Ruthenium Red and with the selective TRPV4 antagonist RN-1734 [32].

In a previous study, it was possible to demonstrate the presence of the TRPV4 channel in human ciliary epithelial cells and the effect, after its stimulation, was an increment of melatonin levels [29]. In fact, in a different study carried out in our lab, it has shown that patients with elevated intraocular pressure actually have higher melatonin levels in the aqueous humor compared to healthy subjects [15]. Moreover, in this study, and supporting the previous observation, it has been possible to visualize the changes in AANAT expression in the ciliary body when comparing normal and glaucomatous individuals (Figure 7). This increase may explain the rise of melatonin in glaucomatous patients and opens the question of why, as melatonin concentrations are abnormally elevated in glaucomatous individuals, this substance cannot reduce IOP as should be expected. In this sense, many studies have reported the hypotensive action of melatonin and analogs either in normotensive and glaucomatous animal models [33,34] as well as in normotensive and ocular hypertensive human beings [12,35]. The reason why the exogenously added melatonin or analogs, produce a reduction in IOP, seems to be related to the dose reached in the aqueous humor. In the glaucomatous patient´s ciliary body and in the human ciliary epithelial cells the elevation of AANAT explains the rise in melatonin in their aqueous humor as if the eye wanted to counteract the IOP by acting on melatonin receptors, as the melatonin is unable to perform its hypotensive effect [15]. More studies are necessary to understand why melatonin cannot reduce IOP in glaucomatous patients.

Glaucoma pathology is known as the silent thief of sight, as it progressively damages the retinal cells. Nevertheless, the main risk factor of this disease is, in fact, the elevated intraocular pressure which can occur either by an increment of the aqueous humor production from the non-pigmented ciliary body cells or by a decrease in its drainage through the uveoscleral outflow pathway or the conventional way through the trabecular meshwork [36]. Previous studies in search of finding new treatments for glaucoma have shown that TRPV4 is linked to trabecular meshwork cells. TRPV4 activation mediates Ca^2+^ influx in the trabecular meshwork which, after the use of an antagonist for this channel, resulted in a decrease in IOP in a murine model of glaucoma [37]. This would imply that the antagonism of the TRPV4 in the trabecular meshwork could protect the retinal ganglion cells from mechanical stress [38,39]. However, different studies have shown the opposite effect on IOP after TRPV4 activation in Lowe syndrome patients. In this sense, TRPV4 activation lowered IOP by acting on the cilia present in the trabecular meshwork [40].

In this study, together with previous ones [15], it has been possible to establish a clear link between melatonin synthesis and the TRPV4, pressure sensor channel, which could lead the ciliary epithelium cells to produce more AANAT in order to synthesize melatonin, which may modify the physiology of those tissues bathed by the aqueous humor.

## 4. Materials and Methods

### 4.1. Cells

Non-pigmented ciliary epithelial cells (59HCE), a human immortalized cell line was kindly supplied by Miguel Coca-Prados. Cells were grown in high glucose Dulbecco’s modified Eagle’s medium (Gibco/Invitrogen, Carlsbad, CA, USA) containing 10% fetal bovine serum (Sigma-Aldrich, St. Louis, MO, USA) and 0.05 mg/mL Gentamicin (Gibco/Invitrogen) at 37 °C in humidified atmosphere 5% CO_2_–95% air. After the culture reached the confluence, cells were detached with 0.25% trypsin and seeded into 6-well plates and/or to 4-well chamber slides, respectively. All the experiments were performed using cells comprising numbers 10–15 passages to assure assays reproducibility.

### 4.2. Human Eye Tissues

Donor Human eyes were obtained from the Fundación Banco de Sangre y Tejidos de las Islas Baleares (Blood and tissue bank Foundation from Baleares Islands). This has been approved by the Ethics Committee of the Universidad Complutense de Madrid with reference C.P.-C.I. 16/249-E (21 June 2016). Six donor eyes were used for this assay, two of a healthy normal subject and another four of glaucoma patients. Eyes were enucleated and collected without the cornea in sterile tubes and maintained in 4% paraformaldehyde in 0.1 M phosphate buffer (PB) (pH 7.2–7.4) at 4 °C until posterior processing. Eyes were dissected under stereomicroscope (Zeiss) and with the 0.8 mm tip curved forceps and sterile dissecting scissors, the iris and ciliary processes were collected. Several washes in phosphate buffer saline (PBS) were performed and then, the specimens were cryoprotected in a sucrose gradient (from 11% to 33%) and were embedded in tissue freezing medium (Tissue-Tek^®^ OCT, Qiagen, Barcelona, Spain) until frozen with liquid nitrogen. Vertical sections of control and glaucomatous human samples (10 µm thick) were collected using a cryostat (Microm, Walldorf, Germany) and mounted from the same region. Samples were maintained in a −20 °C until use.

### 4.3. Immunofluorescent Studies

Frozen sections were rinsed in PBS 1X and permeabilized with PBS-0.05% Tx-100 solution for 30 min. Afterwards, to avoid non-specific staining, sections were incubated in a blocking solution with a 10% normal donkey serum (NDS, Jackson Immunoresearch, West Grove, PA, USA) during 1 h at room temperature. Then, the primary antibody rabbit anti-serotonin *N*-acetyltrasnferase (AANAT, ab3505, Abcam, Cambrigde, UK) was incubated at a 1:500 dilution at 4 °C overnight. Sections were washed in PBS1X-0.1% Tx-100 and incubated with donkey anti-immunoglobulin IgG rabbit antibody conjugated with fluorescein isothiocyanate (FITC; green, Jackson ImmunoResearch, West Grove) at 1:100 dilution in PBS-0.1% Tx-100 for 1 h in a dark chamber at a room temperature. The nuclei were stained with propidium iodide (red, Sigma-Aldrich, St. Louis, MO, USA) diluted 1:500 in PBS for 10 min. Finally, sections were rinsed and mounted in Vectashield (Vector Laboratories, Palex Medical, Barcelona, Spain) and coverslipped. The samples were examined under a confocal microscope (Zeiss LSM 5, Jena, Germany) at 40× magnification. For ciliary epithelial cells immunostaining, similar protocol was done for immunostaining of ciliary epithelium cells after incubating them with TRPV4 agonist GSK1016790A using a rabbit anti-TPRV4 (ab94868, 1:1000 Abcam) primary antibody.

### 4.4. TRPV4 Experiments

Non-pigmented ciliary body epithelial cells were seeded in multiwells of 6 at a density of 1.2 × 10^6^ cells and then treated with the TRPV4 agonist GSK1016790A (Tocris Bioscience, Bristol, UK), for different durations starting from 1, 3, 6, 18, 24, and 48 h to establish a time course at a concentration of 10 nM. The supernatants at the indicated times were collected for melatonin quantification by HPLC as described below. After the corresponding times cells were submitted to lysis as described below for Western blot assays.

In different multiwells, after choosing the best time to see an increase of AANAT, a dose–response curve was obtained by incubating the cells with GSK1016790A at different concentrations ranging from 1 nM to 10 µM, for 18 h (according to the time-course results). Then, cell lysis was performed to quantify AANAT (see below).

For antagonists studies, the compounds ruthenium red (RR) and RN-1734 (RN) [29], were assayed at concentrations of 1 nM and 10 nM respectively, either alone or 30 min before the application of the TRPV4 agonist GSK1016790A. The supernatants at the indicated times were taken for melatonin quantification by HPLC as described below, as well as lysed cells for AANAT detection.

### 4.5. AANAT Western-Blot Studies

Cells were removed and homogenized in ice with RIPA buffer (1:5 *v*/*v*) containing 50 mM HEPES, pH 8, 150 mM NaCl, 1% NP-40 (*w*/*v*), 0.5% sodium deoxicolate, 0.1% SDS and Halt Protease and Phosphate Inhibitor Cocktail (Thermo Fisher Scientific, Madrid, Spain). The lysates were centrifuged at 15,000× *g* for 15 min at 4 °C. The supernatant was stored at −20 °C until use.

Protein concentration was determined by Pierce BCA Protein Assay Kit (Thermo Fisher Scientific, Madrid, Spain). Cell samples (60 µg proteins) were diluted in Laemmli´s sample buffer, loaded on a 15% SDS-PAGE gels and transferred to nitrocellulose membrane. Blots were blocked with 5% non-fat dry milk (Bio-Rad, Madrid, Spain) for 1 h at room temperature and then they were incubated overnight at 4 °C in TBS1X 0.1% Tween 20 containing 5% non-fat milk (Bio-Rad) (blocking buffer) and AANAT primary antibody (ab3505, 1:1000, Abcam). Mouse monoclonal glyceraldehyde-3-phosphate dehydrogenase (anti-GAPDH 1:500) (Santa Cruz, Dallas, TX, USA) served as a loading control. Membranes were incubated with a goat anti Ig G-rabbit or a goat anti-Ig G mouse conjugated with horseradish peroxidase secondary antibody (Jackson ImmunoResearch, West Grove, PA, USA) for 1 h at room temperature. Then, proteins were revealed by chemiluminiscence using enhanced chemiluminiscence (ECL) detection (Amersham Phamacia Biotech, Barcelona, Spain). Films were scanned with Gel Logic 200 Imaging System (Kodak). The densitometric analysis was performed by using Kodak Molecular Imaging software (v 4.0.3, Kodak, Rochester, NY, USA). The densitometry values of each sample were normalized to respective densitometric GAPDH values.

### 4.6. HPLC Analysis

*N*-acetyl serotonin measurements were performed by HPLC following the protocol described by Alkozi and co-workers [28]. Before injection, the supernatants were heated in a 98 °C dry bath for 2 min before being transferred to ice for 10 min. To eliminate proteins, tubes were centrifuged at 13,000× *g* for 10 min at 4 °C. The analysis by HPLC to detect NAS was carried out using a SunFire18 column (5 μ, 25 cm in length, 0.4 cm inner diameter) from Waters (Milford, MA, USA) equilibrated with a mobile phase consisting of 15% acetonitrile, 0.1% acetic acid and at a flow rate of 0.75 mL/min, detecting NAS at the wavelength of 244 nm.

Melatonin measurements were carried out by HPLC as previously described. Briefly, the HPLC was connected to column Kromaphase C18 with 5.0 μm particle (25 cm in length, 0.4 cm inner diameter) from Scharlau, Madrid, Spain. The HPLC consisted of a 1515 Isocratic HPLC pump, a 2487 dual absorbance detector, and a Reodyne injector, ruled by the program Breeze from Waters (Milford, MA, USA). The mobile phase was obtained with 40% methanol, 60% H_2_O. Chromatograms were obtained at a flow rate of 0.8 mL/min measuring melatonin at a wavelength of 244 nm [27,28].

Quantification of NAS and melatonin was performed by comparing the samples with external standards provided by Sigma (St. Louis, MO, USA).

### 4.7. Statistical Analysis

The data represent the mean ±SEM of 4–6 independent experiments (indicated in each case). Statistical significance was calculated by student *t*-test or *ANOVA* test when necessary. GraphPad Prism, v 5 for MAC (GraphPad Software Inc., San Diego, CA, USA) was used to obtain the plots and to fit nonlinear regression curves in order to obtain the pD_2_ value (EC_50_).

## 5. Conclusions

TRPV4 channel activation, which is present in the human ciliary body and ciliary body epithelial cells, increases the expression of the enzyme AANAT, which elevates the concentration of NAS and melatonin. Elevated intraocular pressure can stimulate this channel too promoting the presence of higher concentrations of melatonin in the aqueous humor of patients with glaucoma as previously observed [15]. Altogether, we can suggest that melatonin and AANAT play an important role in pathologies related to intraocular pressure, although more research is necessary to fully understand the role of melatonin in the homeostasis of the aqueous humor.
